# A pragmatic evidence-based clinical management algorithm for burning mouth syndrome

**DOI:** 10.4317/jced.54247

**Published:** 2018-04-01

**Authors:** Yohanan Kim, Timothy Yoo, Peter Han, Yuan Liu, Jared C. Inman

**Affiliations:** 1 MD, Department of Otolaryngology – Head and Neck Surgery, Loma Linda University Medical Center, Loma Linda, CA, USA; 2 BS, Loma Linda University School of Medicine, Loma Linda, CA, USA

## Abstract

**Background:**

Burning mouth syndrome is a poorly understood disease process with no current standard of treatment. The goal of this article is to provide an evidence-based, practical, clinical algorithm as a guideline for the treatment of burning mouth syndrome.

**Material and Methods:**

Using available evidence and clinical experience, a multi-step management algorithm was developed. A retrospective cohort study was then performed, following STROBE statement guidelines, comparing outcomes of patients who were managed using the algorithm and those who were managed without.

**Results:**

Forty-seven patients were included in the study, with 21 (45%) managed using the algorithm and 26 (55%) managed without. The mean age overall was 60.4 ±16.5 years, and most patients (39, 83%) were female. Cohorts showed no statistical difference in age, sex, overall follow-up time, dysgeusia, geographic tongue, or psychiatric disorder; xerostomia, however, was significantly different, skewed toward the algorithm group. Significantly more non-algorithm patients did not continue care (69% vs. 29%, *p*=0.001). The odds ratio of not continuing care for the non-algorithm group compared to the algorithm group was 5.6 [1.6, 19.8]. Improvement in pain was significantly more likely in the algorithm group (*p*=0.001), with an odds ratio of 27.5 [3.1, 242.0].

**Conclusions:**

We present a basic clinical management algorithm for burning mouth syndrome which may increase the likelihood of pain improvement and patient follow-up.

** Key words:**Burning mouth syndrome, burning tongue, glossodynia, oral pain, oral burning, therapy, treatment.

## Introduction

Burning mouth syndrome (BMS), an idiopathic condition characterized by chronic oral mucosal burning and pain, afflicts between 0.7 and 7% of the population (1-3). Often the disease occurs in peri- or post-menopausal women between 50 and 70 years of age (4). Symptoms of the disease include burning or itching of the oral mucosa as well as dysgeusia, paresthesia, dysesthesia, and xerostomia (1). The BMS diagnosis is based on the exclusion of any potential local or systemic causes of burning mouth sensation. Notably, BMS is associated with psychiatric disorders such as depression and anxiety, which have been implicated in potential neuropsychiatric etiologies of the disease (3).

Symptoms of BMS are nonspecific and the diagnosis is often difficult to establish. Furthermore, there are no standard guidelines for management of BMS and physicians are left to employ whatever treatments they are comfortable and/or have experience with. Studies have not revealed any treatments that have been proven to be effective enough to be considered standard of care. Therefore, we sought to address this lack of guidance by developing and testing an algorithm incorporating the various treatment strategies utilized in studies conducted over the last 20 years (5). Our goal was to achieve a balance of efficacy, risk, and practicality.

## Material and Methods

Management Algorithm Development

**Figure 1:**
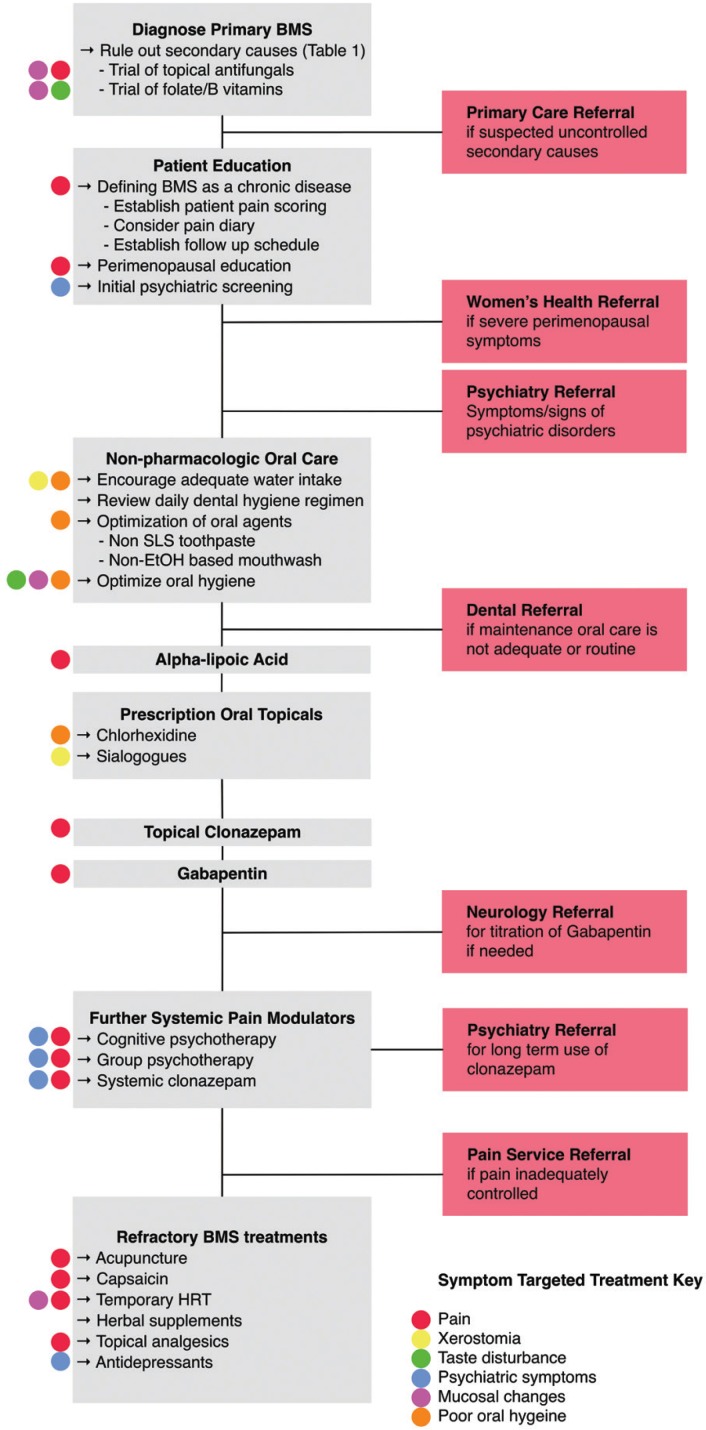
Burning mouth syndrome management algorithm flowchart.

This study was approved by the institutional review board from Loma Linda University Medical Center and appropriate consents were obtained. The development of the evidence- based treatment algorithm we used in this study is supported by the research conducted by Liu *et al.* Below is a summary of each step of the algorithm, which was founded on previous literature and supported by our recent systematic review (5). These steps were taken progressively to achieve adequate symptom control. A flowchart of the algorithm is presented in Figure 1.

Step 1: Diagnosis of Primary BMS

- Thorough history and physical to differentiate from secondary causes of BMS, such as medication(s), fungal infection, and other mucosal diseases (6).

- Once primary BMS is established, start empiric B vitamins, folate, and/or antifungals (7,8).

Step 2: Patient Education

- Educate patients on nature of BMS and provide reassurance (9).

- Counsel relevant patients on possible relationship with menopause (10).

- Psychiatric screening and offer psychiatric referral if pertinent.

Step 3: Oral Hygiene Optimization

- Encourage oral hygiene standards per current American Dental Association recommendations, including (1) brushing teeth twice daily for two minutes each time, (2) tongue brushing, (3) daily flossing, and (4) regular dental visits for professional cleaning and oral exams (11).

- Trial non-alcoholic mouthwashes and avoid foaming agents in toothpaste such as sodium lauryl sulfate (SLS).

- Prevent xerostomia by promoting adequate fluid intake; avoidance of alcohol, tobacco, overly salty foods, acidic beverages, and consumption of sugar-rich foods (12).

Step 4: Alpha-lipoic Acid Supplementation

- ALA trial regimen of 600mg/day for 3-4 months with consideration for treatment extension if improvement but relapse in symptoms after discontinuation (13).

- If symptoms fail to improve sufficiently, gabapentin or psychotherapy may be added, as both have been shown to work synergistically with ALA (5).

Step 5: Prescription Oral Topical Medications

- Trial of daily chlorhexidine gluconate (14).

-Increase salivary flow with over-the-counter artificial saliva (lozenges, rinses, sprays, and swabs) and sialagogues (sugarless gum/lozenges, muscarinic agonists) (15).

Step 6: Neuropathic Treatments

- Clonazepam can be used topically using 1 mg tablets held intraorally near the sites of pain for 3 minutes without swallowing, followed by expectoration, three times a day (16).

- Gabapentin may also be considered, starting at 300mg/day, with gradual titration up to 2400mg/day if necessary (17).

- Consider neurology consultation for long-term use and titration due to potential adverse neurologic effects with extended use.

Step 7: Psychiatric Consultation and Treatment

- Patients are usually not initially inclined to obtain psychiatric consultation. It may be offered again at this time given multiple treatment failures.

- Consider psychotherapy.(5)

Step 8: Systemic Clonazepam

- Trial clonazepam 0.5 mg daily for 9 weeks (18).

- Consider psychiatry consultation for long-term use.

Step 9: Treatment of Refractory BMS

- Consider antidepressants in patients with comorbid psychiatric disease (3).

- Consider hormone replacement therapy (HRT) along with consultation with women’s health (10).

- Consider less-studied and equivocal modalities such as benzydamine hydrochloride oral rinse (19), sucralfate oral rinse (20), topical capsaicin rinse (21), catuama herbal supplementation (22), acupuncture (23), or lycopene enriched olive oil (24).

Analysis of Algorithm Effectiveness

A retrospective cohort study was conducted for 2013 to 2016 comparing all patients who underwent BMS management using the algorithm described above with those who did not. The STROBE Initiative statement was followed (25). All patients were tertiary consults to the Department of Otolaryngology and were treated using the algorithm or without a specific algorithm in accordance with consultants own training and knowledge. Patient records were obtained from a broad electronic medical record review using the broad diagnostic codes such as 529.6 (glossodynia) and 529.1 (geographic tongue), as there is no formal “burning mouth syndrome” code. The patient’s charts were then reviewed to ensure that the patients met the literature or research definition of primary BMS criteria previously outlined in the introduction. All patients diagnosed with primary BMS were included for analysis. Pain was evaluated by documentation of patient pain in the subjective history section and the nursing documentation. Demographics and BMS related data were extracted and patient groups were analyzed. Bias was addressed by having two independent reviews (Y.K. and T.Y.) of the patient charts and data collection with discrepancies resolved by a third independent review from the senior author (J.I.).

The two-tailed, unequal variance, Student t-test and Fisher exact test were used where appropriate to compare groups. Means are reported as mean ±standard deviation (SD). Odds ratios (OR) and confidence intervals (CI) are reported as OR [lower 95% CI, upper 95 % CI]. Significance was established at the *p*<0.05 level (sample sizes were adequately powered, beta 0.2, to achieve improved pain versus no change pain differences and for continued follow-up on the algorithm versus did not follow-up).

## Results

A total of 47 patients were met the appropriate criteria and were diagnosed with primary BMS. Of those patients, 21 (45%) were managed using the algorithm and 26 (55%) were not. Patient characteristics are presented in  Table 1. The mean age overall was 60.4 ±16.5 years, and most patients (39, 83%) were female, 8 or 17% were male. The algorithm group was not significantly different from the non-algorithm groups in terms of age, sex, or follow-up time. However, significantly more algorithm patients had xerostomia (62% vs. 8%, *p*<0.001), and significantly more non-algorithm patients did not continue care (69% vs. 29%, *p*=0.001). The OR of not continuing care for the non-algorithm group compared to the algorithm group was 5.6 [1.6, 19.8].

**Table 1:**
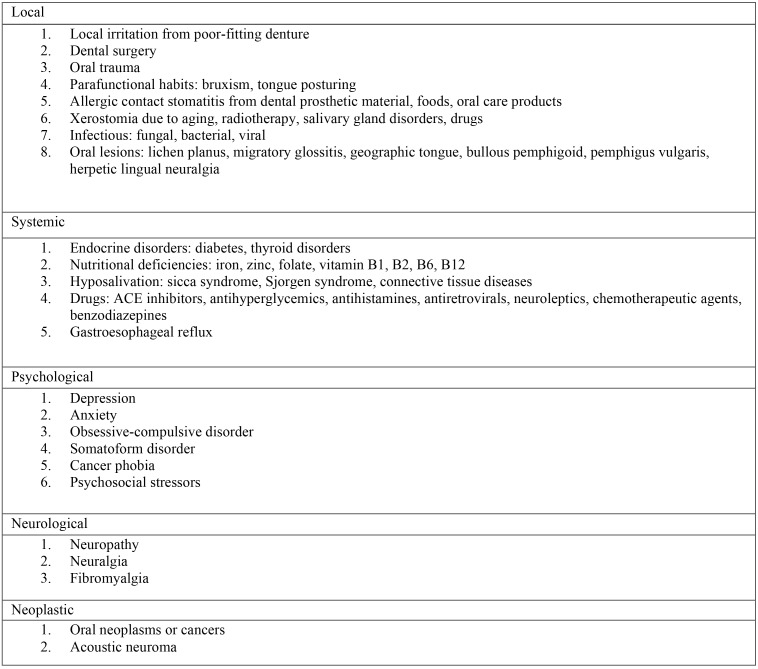
Secondary, contributory, or associated causes of burning mouth syndrome (3).

Improvement in pain was significantly more likely in the algorithm group (*p*=0.001). The distribution of pain outcomes is shown in  Table 2. The OR of experiencing an improvement in pain after undergoing the algorithm-based management versus the non-algorithm management was 27.5 [3.1, 242.0].

**Table 2:**
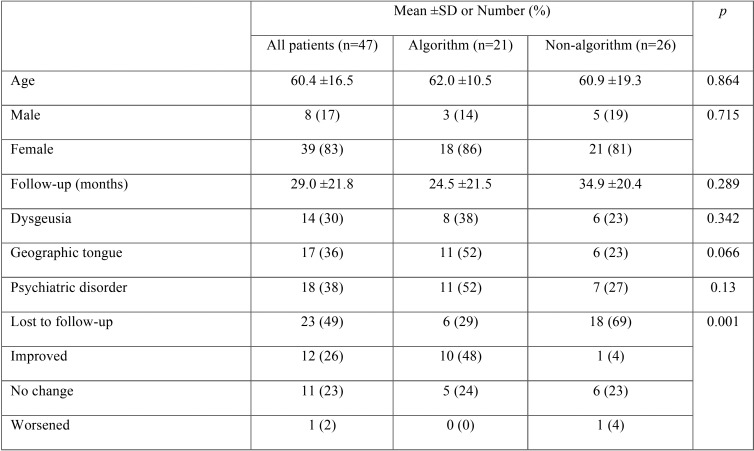
Patient characteristics and distribution of outcomes comparing patients managed with the algorithm and those managed without. *P*-values indicate comparison between the algorithm and non-algorithm groups. The last four rows indicate change in pain/burning symptoms from first to last visit.

## Discussion

Burning mouth syndrome is a poorly understood condition that frustrates both the patient and the physician. Treatments are varied and no one modality stands above the rest (5,26). Moreover, initial treatments are often ineffective and evidence shows that this disease is a chronic pain disorder with a highly variable treatment course. No standardized treatments or guidelines exist, and as such, BMS is a very difficult disorder to treat with its primary symptom being one of chronic pain. This was the impetus for our research, to use the existing knowledge of treatment efficacy to develop a pragmatic, clinically useful management algorithm.

The underlying principle of our algorithm was essentially to rank treatment modalities by balancing efficacy with adverse effects and accessibility by using the best available evidence and the literature and clinical experience (5). It is a stepwise ladder that starts with proper diagnosis and ends with management of refractory cases. It is important to note that the order in which we placed different therapies on the ladder was based primarily on evidence from RCTs, then other evidence levels in the literature, and lastly on our own experience. Therefore, other physicians, dentists, and oral health providers may have differing opinions about which treatments belong where on the timeline of treatment. Furthermore, clinicians may have anecdotal evidence from their own experience to reposition certain treatments or even add in other modalities. In treating this complex, frustrating, and multifactorial chronic disease while going through the algorithm, it is usually helpful to consider consultations with other specialties, such as primary care, psychiatry, dentistry, and women’s health to obtain assistance with medication usage, counseling, and other aspects of management. The exact timing at which consultations are obtained is less important than delivery of adequate care such that patients have sufficient support to understand and continue managing their own illness.

Another principle we attempted to follow in our algorithm was to incorporate different classes of medications/therapies to tackle the symptomatic presentation in each BMS patient, as opposed to using 2 or 3 treatments (e.g. antifungals, vitamins, prescription mouthwashes) for every patient that is typical of non-algorithmic approaches.

We found a significant difference in the rate of improvement in patients who underwent the algorithmic management (48%) from those who did not (4%). Overall, patients in our study were similar to those found in the literature, with a majority of perimenopausal females and symptom/comorbidity frequencies similar to that found in other studies (10). The baseline characteristics of the 2 cohorts were similar except for the rate of xerostomia. We found that unless xerostomia was specifically questioned by the physician it most often was left out of patient subjective history component of the presentation, possibly due to the focus on the pain component of this disease process. We found, when xerostomia was specifically screened that it was more prevalent in the algorithm group (62% vs. 8%). This actually lends support to our management algorithm’s success in that patients with xerostomia are typically more difficult to treat when it is an associated finding in BMS pain. Xerostomia is a comorbid sign and symptom, if recognized, in subsets of BMS cohorts, and is present when directly studied in 46-67% of patients. One explanation for this difference is that the presence of xerostomia may not have been recognized in the non-algorithm group—as it was not directly elicited and was only documented when it was a patient complaint—whereas it was routinely asked in the algorithm group with the help of the patient history survey we use, which was discussed earlier in the methods. Further standardized prospective studies will help better delineate associated findings, like xerostomia, and their effect on BMS symptom severity and on treatment response; however, in order to establish these studies, physicians and researchers need to standardize patient symptom and sign collection relevant to BMS patients specifically (5).

There was significantly more attrition in the non-algorithm group, with 69% of patients failing to continue care compared to 29% in the algorithm group. This may be due to better patient education in the algorithm group which strengthens the patient-physician relationship and reduces “doctor shopping.” We want to emphasize that indeed, treating BMS is a time-consuming and drawn-out process, given the natural history of the disease and its progression over months to years (3). Patients, by natural history of this chronic disease process, will likely have symptoms for years and, moreover, treatment success requires optimization of co-morbid symptoms and frequently “trials” of medications over time. Thus, an expectation for follow up and setting a follow-up schedule would be consistent with management of this chronic disorder. We have attempted to limit attrition by using our standardized patient education tool which was discussed earlier in the methods and by ensuring follow-up appointments for symptom assessment and treatment response are planned out in regular time intervals, similar to other chronic pain syndromes or spectrums of disease.

Our study is limited by the number of total patients; however, it compares favorably to the existing literature cohorts of BMS patients. Another limitation is the number of patients who failed to continue care under our practice, a consistent finding in chronic pain patients. Prospective, clinical treatment strategies, emphasizing follow up through patient education and stepwise treatment trials are needed in this chronic pain disorder. There are intrinsic biases with retrospective studies and prospective trials with a stepwise treatment foundation would be of benefit. Acknowledging this study’s limitations, we hope this basic algorithm would serve as a practical start to the development of more comprehensive management guides. With the advent of new medications, stronger studies, and better understanding of this chronic pain disease process we propose a more algorithmic approach focusing on a stepwise treatment strategy and emphasizing proper patient education and follow-up.
